# Descriptive Epidemiology in Mexican children with cancer under an open national public health insurance program

**DOI:** 10.1186/1471-2407-14-790

**Published:** 2014-10-29

**Authors:** Roberto Rivera-Luna, Jaime Shalkow-Klincovstein, Liliana Velasco-Hidalgo, Rocio Cárdenas-Cardós, Marta Zapata-Tarrés, Alberto Olaya-Vargas, Marco R Aguilar-Ortiz, Eduardo Altamirano-Alvarez, Cecilia Correa-Gonzalez, Fernando Sánchez-Zubieta, Francisco Pantoja-Guillen

**Affiliations:** Head of the Division of Pediatric Hem/Oncology, National Institute of Pediatrics (NIP), Coordinator for the Technical Committee of the National Council for the Prevention and Treatment of Childhood Cancer, Mexico City, Mexico; Program of the National Council for the Prevention and Treatment of Childhood Cancer- CENSIA, Mexico City, Mexico; Department of Pediatric Oncology, NIP, Mexico City, Mexico; Department of Oncology, NIP and Regional Coordinator from the Program of the National Council for the Prevention and Treatment of Childhood Cancer, Mexico City, Mexico; Bone Marrow Transplant Program of the National Council for the Prevention and Treatment of Childhood Cancer, Mexico City, Mexico; Department of Pediatric Oncology NIP, Program of the National Council for the Prevention and Treatment of Childhood Cancer- CENSIA, Mexico City, Mexico; Department of Pediatric Oncology, General Hospital and National Council for the Prevention and Treatment of Childhood Cancer, La Paz Baja California South, Mexico; Department of Pediatric Oncology, Central Hospital “Morones Prieto” and Program of the National Council for the Prevention and Treatment of Childhood Cancer, San Luis Potosi, Mexico; Department of Pediatric Hem/Oncology, Hospital Civil Guadalajara “Juan E. Menchaca and Program of the National Council for the Prevention and Treatment of Childhood Cancer, Guadalajara, Jal Mexico; Pediatric Oncology Department, Hospital General “Dr. Agustin O’Horan and Program of the National Council for the Prevention and Treatment of Childhood Cancer, Merida Yuc, Mexico

**Keywords:** Childhood cancer, Incidence, Prevalence, Mortality, Mexican children

## Abstract

**Background:**

All the children registered at the National Council for the Prevention and Treatment of Childhood Cancer were analyzed. The rationale for this Federal Government Council is to financially support the treatment of all children registered into this system. All patients are within a network of 55 public certified hospitals nationwide.

**Methods:**

In the current study, data from 2007 to 2012 are presented for all patients (0–18 years) with a pathological diagnosis of leukemia, lymphoma and solid tumors. The parameters analyzed were prevalence, incidence, mortality, and abandonment rate.

**Results:**

A diagnosis of cancer was documented in 14,178 children. The incidence was of 156.9/million/year (2012). The median age was 4.9. The most common childhood cancer is leukemia, which occurs in 49.8% of patients (2007–2012); and has an incidence rate of 78.1/million/year (2012). The national mortality rate was 5.3/100,000 in 2012, however in the group between 15 to 18 years it reaches a level of 8.6.

**Conclusions:**

The study demonstrates that there is a high incidence of childhood cancer in Mexico. In particular, the results reveal an elevated incidence and prevalence of leukemia especially from 0 to 4 years. Only 4.7% of these patients abandoned treatment. The clinical outcome for all of the children studied improved since the establishment of this national program.

## Background

Of the biggest problems in developing nations is the high incidence of childhood cancer is the high incidence and the limited financial resources to develop a solid national program for early diagnosis and management of these patients. We believe that pediatric cancer should now be considered a global child health priority [1].

A worldwide childhood cancer survey in 2012 estimates a higher incidence in developing countries (147,000 cancers/year) than in developed nations [2]. It is expected that this disease will continue growing because the populations in these countries are younger and expanding.

Since 2010 in Mexico, cancer is the second leading cause of mortality among children between 4 to 15 years old [3]. This represents a current national health problem. There is a need to expand, fortify and improve early diagnosis and treatment. As we have previously documented in developing countries including Mexico, there is increasing evidence that the mortality rate is higher than in developed countries [4]. This might be related to a late referral to expert institutions for diagnosis and early multidisciplinary treatment.

Close to half of the Mexican-children and adults-are under a medical socialized program. This system contains large hospitals and clinics nationwide and has been run by the Federal Government Socialized Medicine Program for more than 50 years. Formal, salaried sector workers and their families had been able to access pooled-prepayment options through public social security programs.

Meanwhile the other half (*n*: 51,823,314) of Mexicans, including children and adults (2011) [4] has been progressively incorporating into a national health insurance program. This universal health coverage is described as “Popular Medical Insurance” (PMI) [5]. This program was developed to provide access to a package of comprehensive health services-including childhood cancer treatment with financial protection. This is done through a national medical network from primary care clinics to specialized medical hospitals certified by the Federal Government. The program officially started in January 2005. However, it was not until 2007 that the full coverage was issued for all childhood cancers [6].

A referral system has been progressively developed among this network to diagnose, treat and follow all of these children. Currently 55 accredited medical institutions nationwide with departments of pediatric hem/oncology are working fulltime. In each institution, there is at least one certified pediatric hem/oncologist. The population enrolled in this program belongs to the lowest socio-economic levels, and their parents are mostly non-salaried workers, rural residents and the unemployed.

The National Pediatric Cancer Registry under the auspices of the Mexican Federal Government was started in 2006 but it was not until January 2007 that a national registry appeared [7]. This registry includes all Mexican children with cancer regardless of the medical system to which they belong.

The purpose of this report is to present the prevalence, incidence, mortality, and rate of abandonment in the PMI program between 2007 and 2012.

## Methods

An analysis was performed from 2007 to 2012 on all the children from 0 to 18 years diagnosed with cancer registered at the PMI program from all of the accredited institutions.

The diagnosis of leukemia was determined through bone marrow aspiration for cytomorphology analysis, immunophenotype, DNA index and cytogenetic. The diagnosis of lymphomas and malignant solid tumors was performed through biopsy and/or surgical resection for pathology diagnosis. All institutional pathologic reports were classified under the International Classification of Childhood Cancer (ICCC-3) [8].

The medical data on each patient was obtained from the National Commission on Health Social Protection (PMI) which is the financial component of the program and from the National Council for the Prevention and Treatment of Childhood Cancer (CENSIA) headquarters which is the technical and normative entity. Both strategies are under the Federal Department of Health, and they work together collaboratively and exchange all of the patient information. This data managing collaboration occurs through an on-line process. The current work is a retrospective, descriptive study at a national level and the data presented was analyzed from the data base program, no approval from the Ethics Committee was required.

The variables obtained included in each patient’s hospital registration: location of the treatment center, name, address, date of birth, age, gender, diagnosis, stage of the disease, follow up during treatment or under clinical surveillance for more than 3 months, date of death and/or abandonment. Only patients registered in this program were included. Patients were subjected to standardized treatment protocol regimens according to their respective diagnosis, and stage. All protocols were evaluated previously by the National Council of Health [9] follow by CENSIA [10]. The chemotherapy treatment protocols were developed through a panel of experts that designated a national coordinator and associate coordinators. Development of this protocols took place from 2004 to 2006. All of the treatment regimens were developed on the basis of international cancer treatment protocols that demonstrated solid therapeutic results. The scope of the current study is to analyze exclusively the epidemiological aspects of this population.

This descriptive work comprises patients from 55 accredited institutions registered from January 2007 to December 2012. The institutional accreditation criteria were applied through a methodical evaluation from the Office of Medical Innovation and Medical Quality of the National Health Department and medical personnel trained for this type of task.

From a strategic standpoint, Mexico was divided into 6 geographical areas so as to have a physician supervising each area. The role of this specialist is to organize, assist and solve all the problems connected to treatment regimens and local institutional problems related to the children with cancer.

Prevalence was defined as the number of children who have been diagnosed with a type of cancer, at a given year and expressed as the percentage of the disease in Mexican children between 0 to 18 years and registered in the period of time of the study [2]. The incidence rate was defined as the total number of newly diagnosed cases per year/the total population under 18 years who were-registered with a Medical Policy at the PMI by 1 million population/year (Table 1) [2]. Age-specific incidence was defined as the total number of cases of a specific age group/the total population registered in that age group by 1,000,000 population/year. The overall mortality rate was determined as an absolute number of deceased patients/ 100,000 persons/year [2].

**Table 1:** **Absolute number of children (0–18 years) registered with a popular medical insurance health policy and newly diagnosed children with cancer/year**

Year of registration	Population registered with a PMI health policy (yearly increment)	New cases/year
2007	15,106,100	2,017
2008	15,281,375	2,229
2009	15,497,831	2,287
2010	15,986,150	2,403
2011	16,146,011	2,571
2012	17,014,321	2,671

All patients presented in this work study had a pathological diagnosis of cancer, and were treated with a minimal follow-up of 48 months at registered pediatric oncology units.

The program started on January 5^th^ 2005 [10], and the number of original participating institutions was 16. At the beginning only acute leukemia was introduced into this program. However all childhood cancers were progressively included, until January 2007 after which all types of childhood cancers were registered and treated under the national protocols. Therefore, the current work includes only cases collected from January 2007 to December 2012. Descriptive statistical analysis was performed using GraphPad Instant version 3.0.

## Results

**Figure 1:**
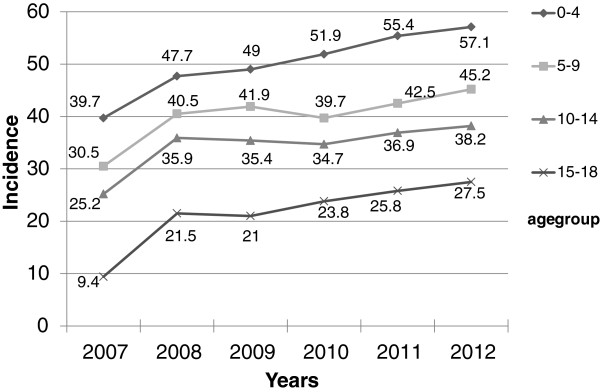
**Incidence by age group during 6 years of the study.**

From January 2007 to 2012 there were 14,178 patients registered with cancer (Table 2). The most common cancer was leukemia (49.8%), followed by lymphoma (9.9%) and central nervous system tumors (9.4%).When the total cohort of children were grouped by age, it showed that the highest incidence was in children between 0–4 years and the lowest was 15 to 18 years through these 6 years (Figure 1). In terms of gender, males were predominately affected [7868 (55.5%)], while the frequency was slightly lower in females [6310 (44.5%)], with a ratio M: F of 1.2:1. Depending upon the type of childhood malignancy males accounted for 54.4% of patients with acute leukemia, 54.2% with brain tumors and 64% of the lymphoma cases (both Hodgkin’s and non-Hodgkin’s subtypes).

**Table 2:** **Prevalence of PMI childhood and adolescent cancers 2007–2012**

Diagnosis	Cases n (%) year 2007	Cases n (%) year 2008	Cases n (%) year 2009	Cases n (%) year 2010	Cases n (%) year 2011	Cases n (%) year 2012	Prevalence 2007–2012 Total n (%)
Leukemia	1,056 (52.4)	1,122 (50.3)	1,133 (49.5)	1,204 (50.1)	1,222 (47.5)	1329 (49.7)	7,066 (49.8)
Lymphomas	207 (10.3)	206 (9.2)	244 (10.7)	247 (10.3)	255 (9.9)	258 (9.6)	1,417 (9.9)
Intracranial neoplasm’s	188 (9.3)	198 (8.9)	198 (8.7)	228 (9.5)	254 (9.9)	277 (10.3)	1,343 (9.4)
Germ cell tumors	53 (2.6)	91 (4.1)	118 (5.2)	146 (6.1)	153 (6.0)	152 (5.6)	713 (5.0)
Soft tissue sarcomas	108 (5.4)	110 (4.9)	95 (4.2)	79 (3.3)	120 (4.7)	112 (4.1)	624 (4.4)
Osteosarcoma	92 (4.6)	104 (4.7)	126 (5.5)	74 (3.1)	94 (3.7)	77 (2.8)	567 (3.9)
Retinoblastoma	75 (3.7)	97 (4.4)	70 (3.1)	93 (3.9)	104 (4.0)	100 (3.7)	539 (3.8)
Renal tumors	80 (4.0)	84 (3.8)	67 (2.9)	89 (3.7)	80 (3.1)	87 (3.2)	487 (3.4)
Miscellaneous reticular neoplasm’s	40 (2.0)	47 (2.1)	58 (2.5)	63 (2.6)	79 (3.1)	95 (3.5)	382 (2.6)
Hepatic tumors	30 (1.5)	44 (2.0)	35 (1.5)	51 (2.1)	67 (2.6)	48 (1.7)	275 (1.9)
Neuroblastoma	39 (1.9)	46 (2.1)	54 (2.4)	38 (1.6)	51 (2.0)	46 (1.7)	274 (1.9)
Ewing tumor and related sarcomas of bone	17 (0.8)	31 (1.4)	35 (1.5)	47 (2.0)	38 (1.5)	24 (0.8)	192 (1.3)
Other solid tumors	32 (1.6.)	49 (2.2)	54 (2.4)	44 (1.8)	54 (2.1)	66 (2.4)	299 (2.1)
Total n cases	2,017	2,229	2,287	2,403	2,571	2,671	14,178

International Classification of Childhood Cancers (ICCC-3)^(7)^.

The national childhood cancer incidence rate in the PMI program over the six years of this study has been increasing (Table 3). In 2012 the incidence was 156.9/million. It was taken into account that the population of Mexico increased from 106,900,000 in 2007 to 113,336,538 in 2012 [3].

**Table 3:** Incidence* of PMI childhood and adolescent cancers

Diagnosis	2007	2008	2009	2010	2011	2012
Leukemia	**69.9**	**73.4**	**73.1**	**75.3**	75.6	**78.1**
Lymphomas	13.7	13.4	15.7	15.4	15.7	15.1
Intracranial neoplasm’s	12.4	12.9	12.7	14.2	15.7	16.2
Germ cell tumors	3.5	5.9	7.6	9.1	9.4	8.9
Soft tissue sarcomas	7.1	7.1	6.1	4.6	4.9	6.5
Osteosarcoma	6.0	6.8	8.1	4.7	6.2	4.5
Retinoblastoma	4.9	6.3	4.5	5.8	6.5	5.8
Renal tumors	5.2	5.4	4.3	5.5	5.0	5.1
Miscellaneous reticular neoplasm’s	2.6	3.0	3.7	3.9	4.9	5.5
Hepatic tumors	1.9	2.8	2.2	2.3	3.1	2.8
Neuroblastoma	2.5	3.0	3.4	3.1	4.1	2.7
Ewing tumor and related sarcomas of bone	**1.1**	**2.0**	**2.2**	**2.9**	2.3	**1.4**
Other solid tumors	2.1	3.2	3.4	2.7	3.3	3.8
Total incidence/year	133.5	145.8	146.2	150.3	159.2	156.9

*Incidence: total number of newly diagnosed cases per year/total population under 18 years by 1,000,000 population/year.

In 2012 the national population census reported 32,972,300 Mexicans between 0 to 18 years (37%). However, only 17,014,321 of the eligible population under 18 years was registered at the PMI program. In the process of tumor classification, Langerhans-cell histiocytosis was included as miscellaneous reticular neoplasm-following the ICCC-3 pathologic classification.

The incidence of leukemia was 78.1/1,000,000/year (2012). The prevalence of leukemia over the six years of the current analysis was 49.8%. The predominance of acute lymphoblastic leukemia was documented in all age groups; (Table 4) in comparison with acute and chronic non-lymphoblastic leukemia. Lymphomas were documented in 1417 patients of which 52.2% had non-Hodgkin’s lymphoma and 47.8% of children presented with Hodgkin’s lymphoma; this difference was not statistically significant.

**Table 4:** **Prevalence of 7066 leukemia’s by age groups registered in the PMI from 2007-2012**

Type of leukemia	0-4	5-9	10-14	15-18	Total
	n	n	n	n	n
	(%)	(%)	(%)	(%)	(%)
Acute lymphoblastic leukemia	2,346(40)	2,170(37)	762(13)	586(10)	5,864(83)
Acute myelogenous leukemia	278(30)	195(21)	185(20)	269(29)	927(13.1)
Chronic myelocytic leukemia	45(23)	54(27)	55(28)	44(22)	198(2.8)
Myelodysplastic syndromes	8(10)	22(28)	24(32)	23(30)	77(1.0)
Total number	2,677	2,441	1,026	922	7,066

This trend clearly indicates that the participation of the PMI accredited institutions and their respective regional coordinators had a beneficial effect on treatment compliance. The national mortality rate/state of residence was from 5.8 in 2007, to 5.3 in 2012/100,000/year. In this last year the lowest rate was in the group under 1 year at 2.5 and was the highest in the group of adolescents between 15 to 18 with a rate of 8.6 (Table 5).

**Table 5:** *Mortality rate by geographical location and by age groups in accredited pediatric cancer institutions in 2012

	***State-rate***	***<1***	***1-4***	***5-9***	***10-14***	***15-18***
***NORTHWEST STATES***	**5.0**					
*1. Baja California*	5.4	5.1	3.3	3.8	5.7	10.0
*2. Baja California South*	6.7	0.0	1.9	11.8	7.7	2.6
*3. Chihuahua*	5.5	4.0	5.4	4.8	2.9	11.6
*4. Durango*	3.1	6.0	2.2	1.2	3.4	4.9
*5. Nayarit*	3.7	4.6	4.4	0.9	4.6	4.6
*6. Sinaloa*	4.5	1.9	3.3	4.0	4.7	6.1
*7. Sonora*	6.6	1.9	6.1	5.5	4.1	12.9
***NORTH EAST STATES***	**4.7**					
*8. Coahuila*	5.1	3.7	6.5	2.6	4.7	6.9
*9. Nuevo León*	4.7	3.5	5.7	3.4	4.1	5.5
*10. San Luis Potosí*	4.4	3.8	1.9	2.2	5.1	8.7
*11. Tamaulipas*	4.9	0.0	5.2	4.7	2.2	9.3
***CENTRAL STATES***	**5.4**					
*12. Aguascalientes*	4.6	0.0	3.9	3.1	3.1	10.8
*13. Colima*	7.1	7.8	8.0	4.8	3.2	13.2
*14. Guanajuato*	5.5	3.5	4.2	4.5	5.1	8.4
*15. Jalisco*	6.3	4.8	5.1	4.6	5.2	11.1
*16. Michoacán*	4.8	5.6	4.2	4.1	2.9	7.8
*17. Querétaro*	5.3	2.7	3.4	5.8	4.2	8.0
*18. Zacatecas*	4.2	3.3	9.0	1.9	1.9	4.4
***CENTRAL METROPOLITAN***	**5.1**					
*19. Hidalgo*	4.8	5.5	3.2	4.8	2.2	9.4
*20. México*	4.7	1.6	4.2	3.8	4.0	7.6
*21. Mexico City*	5.9	0.8	4.8	5.4	4.8	9.6
*22. Tlaxcala*	5.2	4.0	3.0	4.8	4.8	8.3
***SOUTHERN STATES***	**5.4**					
*23. Morelos*	5.2	5.9	1.5	5.3	6.4	5.7
*24. Oaxaca*	5.4	3.7	5.9	4.2	4.6	7.1
*25. Puebla*	5.5	0.8	3.8	4.4	5.5	9.3
*26. Veracruz*	5.7	1.4	3.4	5.0	4.3	11.9
***SOUTHEAST STATES***	**5.7**					
*27. Campeche*	6.1	6.1	3.0	2.4	12.3	4.0
*28. Chiapas*	5.7	1.8	7.8	3.5	3.8	9.1
*29. Guerrero*	3.9	4.1	4.4	2.4	3.1	5.8
*30. Quintana Roo*	5.7	0.0	7.7	5.0	4.5	6.2
*31. Tabasco*	7.7	2.2	8.8	8.8	4.9	8.2
*32. Yucatán*	5.1	0.0	4.6	4.9	3.2	8.7
***NATIONAL RATE***	**5.3**	**2.5**	**4.7**	**4.2**	**4.3**	**8.6**

*mortality rate by 100,000 children/year.

The percentage of patients who abandoned treatment was 4.7%/year, the range was from 4.5% in 2012 to 5.2% in 2007.

## Discussion

The incidence of cancer in children under 18 is increasing especially in developing countries including Mexico [11–14]. To address this problem, several key initiatives have been established by the Mexican Federal Government, including the development of the PMI which applies a diagonal approach to health insurance. Horizontal, population-based coverage is provided for all public and community health services. A package of essential health services is managed at the state-level for all those enrolled with the PMI [5].

The initiatives of this program involve supporting hospital accreditation and providing financial assistance to each institution for the treatment of qualifying children with cancer. These initiatives [14] also include the endorsement of treatment protocols/guidelines, the supply of hospital equipment to pediatric oncology units, technical and financial support for pediatric oncology training programs [13]. By 2011, only 92/150 (61.3%) pediatric hem/oncologists in Mexico were working in accredited hospitals under the PMI program. Rigorous evaluation processes have been underway since the PMI was established, and the results are encouraging for childhood cancer. Adherence to treatment prior to the PMI program was 48% in 2007 and by 2012 rose to 95%.

There were 35,591 children treated for cancer among the 55 PMI accredited Mexican institutions during the period of analysis, which included new patients (14,178) plus 21,413 follow-up patients by the end of 2012. That being said, a pediatric oncologist in the PMI program treated an average of 392 patients in 6 years. In institutions that have one pediatric oncologist for an entire state, the burden of the patient load increases significantly with the imperative to provide a continuously high standard patient care. However, few institutions in Mexico have as many as 8 pediatric oncologists on their full-time staff. This national patient load is obviously higher than recommended for develop countries.

In our current study, we report the findings from a larger number of patients from the Mexican PMI program than the previous study [4]. This represents approximately more than half from the national cancer registry including socialized medicine with their hospitals and in the other side the PMI program. The number of children with cancer registered in the PMI program is very substantial and merits further analysis, especially if these children belong to the lowest socioeconomically deprived bracket of Mexicans. The national childhood cancer incidence [7] in Mexico in the year 2010 from the PMI patients plus those registered in socialized public healthcare systems accounted for 4653 patients with an incidence of 145.5/million/year. The previously published SEER data from the United States [15] has indicated an incidence of childhood cancer (0–19 years) of 173.4/million person/year. In our current study, we have found a higher incidence in childhood cancers than previously published [4] which indicate the need for a more vigorous national public health program for this disease [1]. The active participation of the federal government, which could include providing additional funds to develop better equipped medical institutions and promoting the training of certified pediatricians to enroll in pediatric oncology fellowship programs, is required.

As outline before [2] the high prevalence of leukemia deserves further research especially with the prevalent and constant exposure of particular groups of children in Mexico and elsewhere to organophosphate-based pesticides [16]. Other factors need to be taken into account, including paternal smoking [17], fertilizers and proximity to oil fields [18] especially around the time of conception. Exposure to early infections constitutes another constant factor that is considered in the possible etiology of acute leukemia of childhood especially in the very young [19]. Another factor that might contribute to the etiology of childhood leukemia is in-vitro fertilization, which is reported to be associated with increased risk of early onset of acute lymphoblastic leukemia in the offspring [20].

According to the Mexican national statistics [3] from 112 million inhabitants, there are 50 million that live below the poverty line. This fact, from the public health point of view, should direct attention to the triangle of poverty, malnutrition and early infections that most likely play a very important role in the etiology and incidence of childhood cancer in the PMI population. Recent studies [21] have suggested that the incidence of cancer among children living in poverty is much higher than in more affluent populations. This deficiency might be a factor that can account for the higher incidence of acute lymphoblastic leukemia in comparison to those children treated by the socialized healthcare systems in Mexico, in which the socioeconomic and cultural level is much higher.

Due to the improvement of national public health measures, the incidence of perinatal diseases, malnutrition, gastroenteritis and pneumonia have decreased the pediatric mortality rate in Mexico very significantly, allowing for children to reach an age which childhood cancer is more common. It is ironic that the improved survival rates in children with these diseases may be a factor in the increased incidence rates of cancer. However, incidence and mortality rate are not necessarily correlated in Mexico, as has been outlined [22], but the standard of medical care is constantly improving and the PMI program has had a very beneficial impact on the prognosis for low-income pediatric cancer patients.

In 2004, the National Council of Health from the Federal Health Department and a group of pediatric hem/oncologists produced technical protocols for treating the most commonly occurring childhood malignancies experienced in Mexico. We formulate 26 treatment protocols based on international treatment guidelines [9], which included multidisciplinary treatment approaches for all childhood cancers. One of the principal achievements of this program is the progressive improvement of the overall survival. We were able to identify 11 states in Mexico that had an overall survival for all childhood malignancies beyond 72% versus 10 states in which the overall survival was less than 65%. The possible explanation for this later finding is multifactorial and includes, limited number of pediatric hem/oncologist in these areas, advanced and complicated disease at diagnosis, some with small, space-restricted units, low number of pediatric oncology nurses, and a lack of full-time pediatric specialists available for the multidisciplinary approach including surgeons, intensive care specialists, and radiotherapists. Those institutions that had the best survival rates are affiliated with university hospitals and at least a residency program in pediatrics and a training program in childhood cancer for the nursing personnel.

Regarding mortality, we have been able to maintain a similar rate from 2007 to 2012. However when compared to other developing countries we need to work harder to decrease the mortality even further [23–25]. A very important finding is the high mortality rate in 2012 among adolescents from 15 to 18 years old. One of the observations we have documented in this respect is that more than half of these patients were very advanced or had unfavorable prognostic signs when they arrived for medical care of their cancer. There are multiple possible explanations for these findings and they deserve to be investigated and reported in future scientific publication.

The need to continue monitoring the treatment protocols and avoiding violation of these regimens could eventually lead to better results and a decrease in the mortality rate. On a collegiate basis the protocols are revised every 6 months among all participating institutions and the Council of General Health of Mexico. Proper modifications are performed in those instances where there is a high rate of relapse and/or complications.

Chatenoud, et al. [26] reported a standardized mortality rate (per 100,000/year) of 6.45 and 5.42 among boys and girls respectively in Mexico in the period from 2005–2006. However, in 2012 we documented a standardized overall mortality rate of 5.3 without a significant difference between genders. We recognize that our mortality rate is higher compared with other Latin-American countries [27–29] in spite of the PMI program. Mexican children (<18 years) with cancer had an average lifespan of 10.8 years and therefore impede living on an average of 59.2 years [6], while in develop countries, the survival rate in childhood cancer is higher and better [30]. It is important to direct our attention to pediatric oncology in developing nations. In most of our Latin-American countries there is a failure to make an early diagnoses and, to a lack of treatments regimens-clinical trials and inadequate health care systems [31, 32]. Another point that deserves to be mentioned is the continuous low rate of abandonment that has been maintained during the six years of this study and up to the present time, consistent with observations that international studies have eradicated this problem [32].

## Conclusion

The national childhood cancer registration in the PMI program denotes a high prevalence and incidence among Mexican children. The current experience demonstrates that the creation of a cooperative treatment group for childhood cancer in a developing nation can be accomplished through the efforts of a multidisciplinary approach. The combination of horizontal coverage of personal health services with a catastrophic fund makes it possible to offer financial protection for childhood cancer, as well as investing in early detection and survivorship care. Our future goal is to continue improving the treatment program and therefore the outcome for these children. We believe that children with cancer worldwide deserve a stronger focus on their needs than they are currently receiving, especially in resource-poor settings.
